# Developments in life expectancy in Germany. Current trends

**DOI:** 10.25646/5873

**Published:** 2019-03-14

**Authors:** Enno Nowossadeck, Elena von der Lippe, Thomas Lampert

**Affiliations:** Robert Koch Institute, Berlin Department of Epidemiology and Health Monitoring

**Keywords:** LIFE EXPECTANCY, GENDER, SOCIAL DIFFERENCES, REGIONAL DIFFERENCES, EUROPEAN COMPARISON

## Abstract

Since the beginning of the 1990s, life expectancy in Germany has increased by 4.2 years among women (to 83.2 years), and by 5.9 years among men (to 78.4 years). This rise is related to the increasing convergence of life expectancy in Germany’s new and old federal states. Recently, life expectancy among women in the new federal states has even risen slightly above the level found in the old federal states. In addition, differences between socioeconomic groups continue to be observed in Germany. Women in the highest income group have a 4.4-year longer life expectancy than women in the lowest income group. Similarly, an 8.6-year difference exists between men in the highest income group and men in the lowest income group. Influenza waves can adversely affect the development of life expectancy in certain calendar years. In comparison to other European countries, Germany has a mid-range life expectancy: the current difference between life expectancy in Germany and Switzerland (the European country with the highest life expectancy) is 2.7 years.

## Introduction

Mortality has a strong impact on demographic change. As an example, a decline in mortality can contribute to population growth. In recent decades, mortality has fallen most sharply among people of high and highest age groups. In fact, this decline in mortality in higher ages is one of the causes behind population ageing, in other words, an increased proportion of elderly and very old people in a population. This is also referred to as ‘ageing from above’, i.e. ageing from the upper end of the age structure. Declining mortality is reflected in increased life expectancy. Since about 1840, record life expectancy, in other words, the highest level of life expectancy in the world in a given year, has grown linearly [1]. But it is not only record life expectancy that is rising; life expectancy is also increasing in various regions and countries throughout the world [2]. This Fact sheet focuses on and describes the current trends in mortality and life expectancy in Germany.

## Indicator

Mean life expectancy at birth is an important summary measure that describes the health state of a population. Mean life expectancy refers to the average number of years of life that a new-born child can be expected to live under the mortality rates of the respective year. In addition, it is also possible to calculate life expectancy for a particular age group (such as people aged 65). The measure of further life expectancy indicates the average number of additional years of life that a person of a particular age can be expected to live (e. g. at the age of 65).

The life expectancy estimates used in this Fact sheet were calculated by the Federal Statistical Office using life tables. Each estimate of life expectancy is based on mortality data from three calendar years and uses a moving average to smooth out short-term fluctuations in the data. The last available life table refers to the three-year period 2015/2017 [3]. These data were taken from Germany’s Information System of the Federal Health Monitoring.

## Results and discussion

Life expectancy has increased significantly in Germany in recent decades [4]. Until the middle of the 20th century, this increase was mainly due to declining mortality among infants, children and young adults. Since then, increasing life expectancy has mainly been driven by declining mortality among older people [5, 6]. Life expectancy has continued to rise over the last 25 years. In the beginning of the 1990s (1991/1993), the average life expectancy among women was 79.0 years and 72.5 years among men; in 2015/2017 it had increased to 83.2 years among women and to 78.4 years among men. This corresponds to an increase of 4.2 years for women and 5.9 years for men. However, small interruptions in the continuous increase in life expectancy can be observed, for example, in the 2013/2015 period. Most recently, women faced a very small decline in life expectancy of 0.02 years (2015/2017). For men, life expectancy increased by 0.05 years during the same period.

The latest life table (which covers the period 2015/2017) shows that life expectancy among women is 4.8 years higher than among men. The phenomenon of women’s longer life expectancy, however, is repeated throughout the world [7] and seems to be partly related to biology. The ‘cloister study’ demonstrated that nuns had a survival advantage of approximately one year over monks despite similar health-related behaviour and living conditions [8]. Consequently, biological causes are assumed to account for about one year of the difference between life expectancies among women and men. Non-biological factors, therefore, can be considered as the main cause of the differences in life expectancy among the general population.

Non-biological factors include aspects such as health-related behaviour and people’s living conditions. The most important factors that need to be considered in terms of health-related behaviour are differences in tobacco and alcohol consumption, physical inactivity, diet, and accident-causing behaviour. With regard to living conditions, differences between women and men in labour force participation, working conditions and income distribution are likely to be of particular significance [9].

Differences in life expectancy have also been observed with regard to social status [9, 10]. Women in the highest income group have a 4.4-year longer life expectancy than women in the lowest income group [11]. Similarly, men in the highest income group have an 8.6-year longer life expectancy than men in the lowest income group. The higher rate of mortality among men may be due to the fact that certain subpopulations have particularly high mortality rates that are associated with their lower socioeconomic status [10, 12].

These social differences in life expectancy are also reflected in differences at the regional level. For example, socioeconomic differences in life expectancy were also identified using an analysis conducted at the district and city level. The life expectancy of women in poorer districts and cities is 1.5-years lower than that of women in the most affluent districts; the difference between men in poorer and affluent districts and cities is even greater at 2.9 years [13].

**Figure 1:**
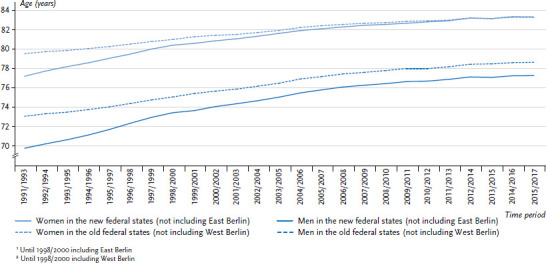
The life expectancy of women and men in the new^1^ and old^2^ German federal states between 1991/1993 and 2015/2017 Source: Federal Statistical Office (2017) Statistics on the natural population change [14]

In the 2014/2016 period, women in the new federal states (former East Germany; excluding East Berlin) had a life expectancy of 83.25 years. This is the first time that women’s life expectancy has been higher in the new federal states than in the old federal states (former West Germany; excluding West Berlin) (83.19 years; Figure 1). This difference also persisted one year later. However, the differences are so small that they could also be due to coincidence.

As early as 2013, a study reported that mortality among women of several age groups in former East and West Germany converged between 2000 and 2009. In the years that followed, mortality in the new federal states in the respective age groups fell below that of the old federal states [15]. It is possible that this process is also reflected in the data on average life expectancy as of the 2014/2016 period.

The 2013 study discussed tobacco consumption as a possible explanation of women’s advantage in mortality in the new federal states. Once causes of deaths that are largely related to tobacco consumption, such as lung cancer, are excluded from the analysis, a higher mortality rate remains in the new federal states. In the beginning of the 1990s, the proportion of female smokers in the old federal states (29%) was higher than in the new federal states (22%). The delay in the onset of tobacco-related diseases can explain the slower increase in women’s life expectancy in the old federal states. However, in recent years, the proportion of smokers in the new federal states has reached a similar level to that in the old federal states [16, 17]. As such, women from the new federal states are expected to lose their advantage in mortality in the future [18].

As pointed out above, the rise in life expectancy has slowed down in Germany in certain years. Influenza waves provide a possible explanation. A number of strong influenza waves have occurred in recent years, such as during the 2012/2013, 2014/2015 and 2016/2017 winters [19]. As influenza outbreaks occur in the first quarter of a year, they are recorded in the second calendar year of a particular winter. Influenza outbreaks often lead to a higher number of deaths than what would normally be expected, a situation known as ‘excess mortality’ [19, 20]. The figures on excess mortality for the winter seasons mentioned above were 20,700 for 2012/2013; 21,300 for 2014/2015, and 22,900 for 2016/2017 [19]. These additional deaths correspond to between 2.3% and 2.5% of the annual deaths that occurred in Germany in 2013, 2015 and 2017, which were precisely the calendar years in which the increase in life expectancy slowed down.

Two influenza waves occurred between 2015 and 2017 – one in 2015, and one in 2017. Therefore, the calculations undertaken for the 2015/2017 life table will have been influenced by the higher excess mortality. The preceding table, which covers 2014 to 2016, only includes one year with an influenza wave (2015). Thus, the impact of influenza outbreaks is likely to be strongest on the 2015/2017 life table. As such, the use of a moving average may have had an impact on the results and slowed down the increase in life expectancy.

The fact that influenza waves can influence mortality in certain calendar years is not a new phenomenon and has already been discussed in population research elsewhere [cf. 21]. In general, however, changes in life expectancy are influenced by many factors. Influenza waves are just one such factor and their effects are of a short-term nature. Other factors, such as medical care, disease prevention, health promotion, rehabilitation and health-related behaviour tend to have a longer term impact.

An international comparison with other European countries shows that Germany has a mid-range life expectancy (Table 1). Switzerland currently has the highest life expectancy in Europe (83.7 years), followed by Spain (83.5) and Italy (83.4). Georgia has the lowest life expectancy in Europe (74.2 years). This places life expectancy in Germany 2.7 years behind the highest level in Europe. In 1991, this difference was 2.3 years. The difference between women’s life expectancy in Germany and in Spain (where women have the highest life expectancy) is 2.8 years; the difference between men’s life expectancy in Germany and Switzerland (where men have the highest life expectancy) is 3.1 years. Since the beginning of the 1990s, Germany has been unable to make up for this gap. Moreover, this difference has even increased somewhat since then, but the reasons for this remain unclear. However, other countries, such as the United Kingdom, the Netherlands, Sweden, Spain and France have also experienced a less pronounced increase in life expectancy in recent years compared to Switzerland [22].

In summary, the increase in life expectancy in Germany is certainly a success. This particularly applies to the situation in Germany’s new federal states. Nevertheless, when the figures are compared to those of other European countries, it is clear that Germany continues to face certain challenges.

## Key statements

- The life expectancy of women in the new federal states is now higher than that of women in the old federal states.
- Life expectancy is rising faster among men than among women.
- The increase in life expectancy has slowed down recently in some calendar years, and a slight drop in life expectancy was observed in 2015/2017.
- Influenza waves could help explain the recent slowdown in life expectancy.

## Figures and Tables

**Table 1:** Life expectancy at birth in selected^*^ European countries according to gender in 1991, 2006 and 2016 Source: Eurostat (2018) [23]

Life expectancy at birth (years)									
Total				Women			Men		
1991		2006	2016	1991	2006	2016	1991	2006	2016
European Union (EU 28)	–	78.9	81.0	–	82.0	83.6	–	75.8	78.2
Austria	75.9	80.1	81.8	79.1	82.8	84.1	72.3	77.1	79.3
Belgium	76.3	79.5	81.5	79.7	82.3	84.0	72.9	76.6	79.0
Bulgaria	71.1	72.7	74.9	74.4	76.3	78.5	68.0	69.2	71.3
Croatia	–	75.9	78.2	–	79.3	81.3	–	72.4	75.0
Cyprus	–	80.1	82.7	–	82.0	84.9	–	78.1	80.5
Czechia	72.0	76.7	79.1	75.8	79.9	82.1	68.2	73.5	76.1
Denmark	75.3	78.4	80.9	78.1	80.7	82.8	72.5	76.1	79.0
Estonia	69.8	73.2	78.0	75.0	78.6	82.2	64.4	67.6	73.3
Finland	75.5	79.5	81.5	79.5	83.1	84.4	71.4	75.9	78.6
France	77.2	80.9	82.7	81.4	84.5	85.7	73.0	77.3	79.5
Germany	75.7	79.9	81.0	78.8	82.4	83.5	72.2	77.2	78.6
Greece	77.3	79.9	81.5	79.8	82.7	84.0	74.8	77.1	78.9
Hungary	69.4	73.5	76.2	74.0	77.8	79.7	65.1	69.2	72.6
Ireland	75.0	79.3	81.8	77.9	81.7	83.6	72.3	76.9	79.9
Italy	77.1	81.4	83.4	80.4	84.1	85.6	73.8	78.6	81.0
Latvia	–	70.6	74.9	–	76.1	79.6	–	65.0	69.8
Lithuania	70.6	71.0	74.9	76.0	77.1	80.1	65.1	65.0	69.5
Luxembourg	75.7	79.4	82.7	79.3	81.9	85.4	72.0	76.8	80.1
Malta	–	79.5	82.6	–	82.0	84.4	–	77.0	80.6
Netherlands	77.2	80.0	81.7	80.3	82.0	83.2	74.1	77.7	80.0
Poland	70.4	75.3	78.0	75.1	79.7	82.0	65.9	70.9	73.9
Portugal	74.1	79.0	81.3	77.7	82.5	84.3	70.5	75.5	78.1
Romania	70.1	72.5	75.3	73.5	76.1	79.1	66.8	69.0	71.7
Slovakia	71.1	74.5	77.3	75.5	78.4	80.7	66.9	70.4	73.8
Slovenia	73.6	78.3	81.2	77.5	82.0	84.3	69.5	74.5	78.2
Spain	77.1	81.1	83.5	80.7	84.4	86.3	73.4	77.8	80.5
Sweden	77.8	81.0	82.4	80.7	83.1	84.1	75.0	78.8	80.6
United Kingdom	–	79.5	81.2	–	81.6	83.0	–	77.3	79.4
Albania	–	–	78.5	–	–	80.1	–	–	77.1
Armenia	–	72.9	75.1	–	76.0	78.4	–	69.7	71.5
Belarus	–	–	74.2	–	–	79.2	–	–	69.0
Georgia	–	74.2	72.7	–	78.4	77.2	–	69.7	68.3
Iceland	78.0	81.2	82.2	81.3	82.9	84.1	74.9	79.5	80.4
Kosovo^+^	–	–	78.6	–	–	81.6	–	–	75.9
Liechtenstein	–	81.0	82.3	–	83.1	84.0	–	78.9	80.6
The former Yugoslav Republic of Macedonia	–	73.9	75.4	–	76.2	77.5	–	71.7	73.4
Montenegro	–	73.9	76.5	–	76.4	78.9	–	71.4	74.1
Norway	77.1	80.6	82.5	80.2	82.9	84.2	74.0	78.2	80.7
Serbia	–	73.4	75.7	–	76.1	78.3	–	70.8	73.2
Switzerland	77.8	81.8	83.7	81.4	84.2	85.6	74.2	79.2	81.7
Turkey	–	–	78.1	–	–	81.0	–	–	75.4

^*^ Only countries with data for 2016

^+^ In accordance with United Nations Security Council Resolution 1244/99
